# Storage of passive motion pattern in hippocampal CA1 region depends on CaMKII/CREB signaling pathway in a motion sickness rodent model

**DOI:** 10.1038/srep43385

**Published:** 2017-02-23

**Authors:** Junqin Wang, Jiluo Liu, Leilei Pan, Ruirui Qi, Peng Liu, Wei Zhou, Yiling Cai

**Affiliations:** 1 Department of Nautical Injury Prevention, Faculty of Navy Medicine, Shanghai, China

## Abstract

Sensory mismatch between actual motion information and anticipated sensory patterns (internal model) is the etiology of motion sickness (MS). Some evidence supports that hippocampus might involve the neural storage of the “internal model”. This study established an “internal model” acquisition-retention behavioral model using a repeated habituation rotation training protocol. We tried to identify the hippocampal subregion involved in “internal model” retention using chemical lesion methods. Hippocampal kinases (CaMK, CaMKIV, CREB and ERK1/2) phosphorylation in the target subregion was assayed and the effects of kinase inhibitors (KN93 or U0126) on “internal model” retention were investigated. The activities of potential kinases (CaMKII and CREB) were also examined in otoliths deficit *het/het* mice. In habituated rats, CA1 lesion reproduced MS-related behavioral responses on “internal model” retention day. Habituation training increased CaMKII and CREB activity but had no effect on CaMKIV and ERK1/2 activity in the CA1, while inhibition of CaMKII but not ERK1/2 impaired “internal model” retention. In *het/het* mice, CaMKII and CREB were not activated in the CA1 on the retention day. These results suggested that CaMKII/CREB pathway might potentially contribute to the storage of the “internal model” in the hippocampal CA1 after motion sickness induced by vestibular stimulation.

Motion sickness (MS) has long been explained by sensory conflict hypothesis which still remains ambiguous regarding the underlying molecular and neurobiology basis. Reason and Brand have demonstrated that the sensory mismatch between the passive motion sensory inputs (vestibular organ, eyes and proprioceptor) and the neural storage of anticipated sensory patterns (internal model) might be the etiology of the spatial disorientation and the autonomic responses of MS^1^. Recently, Cullen and his colleagues have identified the vestibular only (VO) neurons and the “unimodal” rostral fastigial nucleus (u-rFN) neurons that only reacted to passive movements (exafferent) but not to anticipated active ones (reafference) in primates^2^. These new evidence strongly supported the notion that the “internal model” might predict the expected proprioceptive inputs and cancel the motion afferences that match the past experiences. However, the precise brain area where the “internal pattern” of the passive motion pattern is produced and stored still remains unclear.

Previous studies have demonstrated that hippocampal lesion can aggravate MS induced by hypergravity in rats, while surgical vestibular cerebellum lesion has no significant effect^3^,^4^. In the past decade, studies on the interactions between the vestibular-autonomic system and the hippocampus have confirmed that the vestibular information can be processed by the hippocampus formation. In rodents, peripheral vestibular lesions completely abolished location-related firing of the place cells in the CA1 region and impaired the spatial memory but not the non-spatial memory performance^5^,^6^. In humans, acquired chronic bilateral vestibular loss caused a significant selective atrophy of the hippocampus and impaired spatial memory^7^. Additionally, bilateral microinjections of the unspecific synaptic blocker into the dorsal hippocampus attenuated the autonomic responses elicited by a restraint stress exposure^8^. A more recent study showed that bilateral microinjections of the NMDA receptor antagonist into the dorsal hippocampus also attenuated restraint stress-evoked autonomic responses^9^. Furthermore, the theta rhythm in the hippocampal CA1 and the dentate gyrus (DG) subregions can be induced during forward–backward translocation and passive rotation in rats. Electrical stimulation of these regions at theta rhythm (8 Hz) also increased heart rate^10^. These results suggested that sensory conflict information conveyed by the vestibular system might possibly be processed in the hippocampus during MS. In addition, prolonged or repeated passive motion exposure or training can establish novel “internal model” leading to MS habituation. Our previous studies described the “internal model” acquisition processes using a repeated daily rotation model which elicited a gradual alleviation of defecation and locomotion hypoactivity in the rats^11^,^12^. Although such MS habituation processes were commonly seen across species from rodents, cats, monkeys to human beings^13^,^14^, the time-course of the “internal model” acquisition under particular stimulus conditions is normally highly related to the pattern of passive motion. Furthermore, when the training stops, the resistance to the specific motion stimulus will maintain several days or weeks indicating a period of the “internal model” retention. Therefore, we hypothesized that the specific sensory pattern of passive motion might be very likely to be stored in the hippocampus with the ability to handle multiple types of memory.

In the hippocampus, the N-methyl-D-aspartate receptor (NMDAR) signalling and NMDAR-dependent synaptic plasticity are essential for proper representation of space and the spatial learming^15^. Calcium entry through NMDARs leads to the activation of calmodulin which triggers the phosphorylation of Ca^2+^/calmodulin-dependent protein kinases (CaMKs)^16^. Among the many CaMKs, CaMKII and CaMKIV play pivotal roles in the process of learning and the formation of memory through induction of the long-term potentiation (LTP)^17^. In mammals, CaMKII comprises multiple isoforms and αCaMKII is the most abundant one in the hippocampus^18^. Germline deletion of the αCaMKII in the hippocampus leads to deficits in spatial and contextual learning and the LTP^19^. The autophosphorylation of αCaMKII is required in the induction of NMDAR-dependent potentiation at hippocampal CA1 synapses and is essential for spatial memory and cued fear memory formation^20^. In addition to CaMKII, genetic deletion or the inhibition of CaMKIV also impaired synaptic plasticity leading to the deficits in fear memory formation and spatial memory retention^21^,^22^. Furthermore, both αCaMKII and CaMKIV are responsible for phosphorylation of cAMP-response element-binding protein (CREB) which is a gene transcription factor required for for plasticity-related stimulation of gene transcription^23^. Numerous studies have demonstrated that CREB is a conserved regulator of long-term memory in a variety of organisms including worms, flies, mice, and humans^24^. Additionally, synaptic NMDAR activation also triggers extracellular signal-regulated kinase (ERK1/2) cascade which influences synaptic plasticity through its regulation on transcription and translation^25^. ERK1/2 is necessary for the formation of long-term memory^26^. All these studies demonstrated that the hippocampal kinases constitute the molecular basis for hippocampus-dependent learning and memory.

In this study, we tried to test the hypothesis that the hippocampus might be involved in the retention of the “internal model” derived from repeated passive motion stimulation. The “internal model” acquisition process was characterized by MS habituation that is achieved using a repeated daily rotation training protocol in rats and mice. MS-related symptoms were assessed by the defecation responses, the balance disturbance and/or the spontaneous hypoactivity. The time course of “internal model” retention period was determined via observing the reappearance of MS symptoms (sensory conflict) after the training stopped in rats. The dorsal hippocampal subregion that might be potentially involved in the “internal model” retention was identified by selective chemical lesions methods in rats. Furthermore, we examined temporal changes in the activity of the hippocampal kinases (CaMKII, CaMKIV, CREB and ERK1/2) in the identified “internal model” retention-related subregion. The roles of the identified kinases were further verified by infusions of kinase inhibitors (KN93 or U0126) into the dorsal hippocampus in rats during the period of “internal model” retention. At last, the B6Ei.GL-Nox3*het (het/het*) mouse strain absent of otoliths was used to investigate the contribution of vestibular afference to the “internal model” retention-related alterations in kinase activity in the hippocampus.

## Results

### The acquisition and retention of a novel “internal model” after Hab training in rats

**Figure 1:**
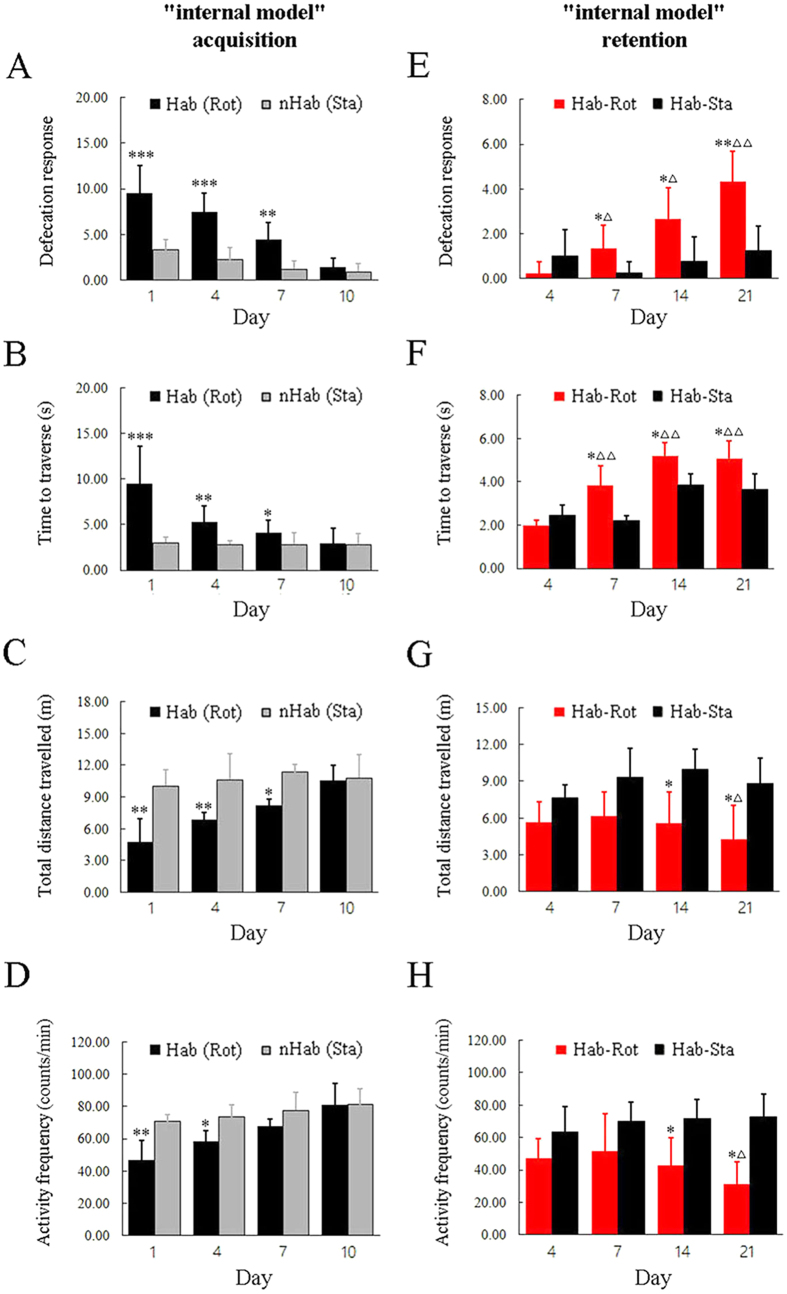
MS-related behavioral responses during “internal model” acquisition and retention period. Defecation responses, time to traverse the balance beam, and total distance travelled and activity frequency in an open field were measured in animals receiving habituation (Hab) or non-habituation (nHab) treatment (**A–D**) and in habituated animals exposed to Rot or Sta on the days after the training has stopped (**E–H**). Data are represented as mean ± SEM. *P < 0.05, **P < 0.01 compared with the corresponding Sta control group. ∆P < 0.05, ∆∆P < 0.01 compared with the Hab-Rot group on Day 4 after the Hab training has stopped.

During the “internal model” acquisition period, animals showed a significant increase in the defecation responses (one-way ANOVA: F_(7, 95)_ = 81.939, P < 0.001; post hoc: P < 0.001; Fig. 1A), a significant increase in the time to traverse the balance beam (one-way ANOVA: F_(7, 47)_ = 39.700, P < 0.001; post hoc: P < 0.001, 0.01 or 0.05; Fig. 1B), and a significant decrease in the spontaneous locomotor activity (total distance travelled: F_(7, 47)_ = 15.176, P < 0.001; post hoc: P < 0.001 or 0.01; Fig. 1C; activity frequency: F_(7, 95)_ = 12.632, P < 0.001; post hoc: P < 0.001, 0.01 or 0.05; Fig. 1D) after receiving 1, 4 or 7 sessions of Rot compared with the corresponding Sta controls. Notably, these behavioral responses showed apparent signs of habituation and there were no difference between the Rot and the Sta animals receiving 10 sessions of treatment (post hoc: P > 0.05), indicating the establishment of a novel “internal model”. During the “internal model” retention period, there were significant differences among the Hab-Rot and the Hab-Sta groups in the defecation responses (F_(7, 63)_ = 8.333, P < 0.001), the balance disorders (F_(7, 63)_ = 11.313, P < 0.001) and the spontaneous locomotor activity (total distance traveled: F_(7, 63)_ = 6.086; activity frequency: F_(7, 63)_ = 4.276, P < 0.001). No significant difference in these MS behavioral indexes was observed between the Hab-Rot and the Hab-Sta group on Day 4 after training (post hoc: P > 0.05; Fig. 1E–H). The defecation responses and the balance disorders reappeared on Day 7, 14 and 21, while the spontaneous locomotor activity reappeared on Day 14 and 21 in the Hab-Rot animals compared with the corresponding Hab-Sta controls (post hoc: P < 0.05 or 0.01). The magnitude of the defecation responses and the balance disorders were also significantly higher in the Hab-Rot groups on Day 14 and Day-21 than on Day 4 (post hoc: P < 0.05 or 0.01; Fig. 1E and F). Both the total distance traveled and the activity frequency were significantly lower in the Hab-Rot animals on Day 21 than on Day 4 (post hoc: P < 0.05; Fig. 1G and H).

### Hippocampal CA1 subregion lesion impaired “internal model” retention in rats

**Figure 2:**
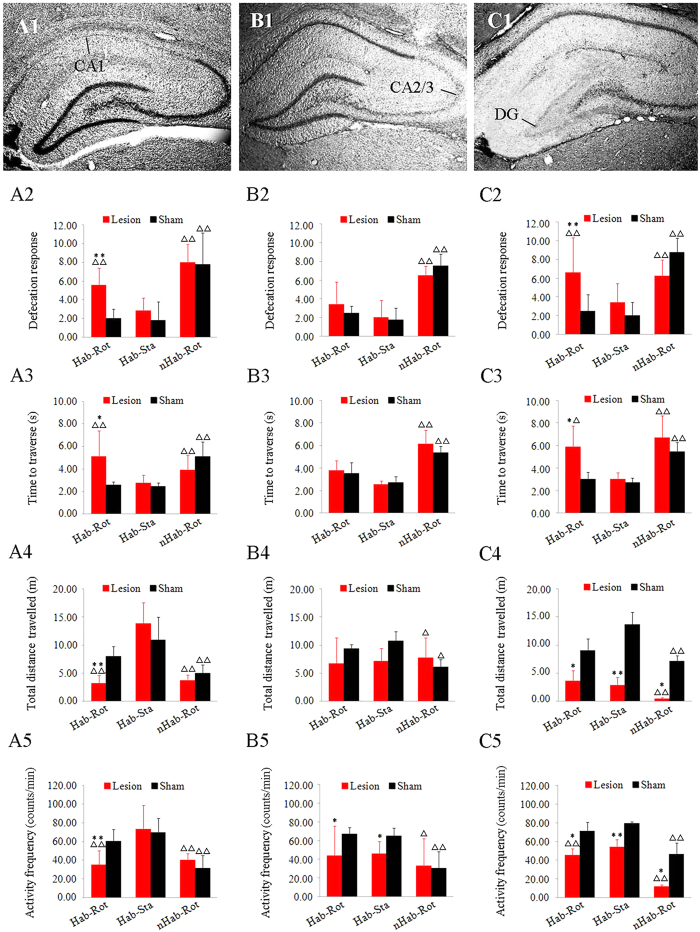
Effects of hippocampal subregion lesion on MS-related behavioral responses during “internal model” retention period. Representative histological images of Nissl staining in animals receiving ibotenic acid injections into CA1 (about 3.6 mm caudal to bregma; A1), CA2/3 (about 3.3 mm caudal to bregma; B1) and colchicine injections into DG (about 4.3 mm caudal to bregma; C1). Defecation responses (A2, B2 and C2), time to traverse the balance beam (A3, B3 and C3), as well as total distance travelled (A4, B4 and C4) and activity frequency (A5, B5 and C5) in an open field were analyzed on Day 4 during habituation retention period in hippocampus-lesioned or sham-operated animals receiving prior Hab training or nHab treatment. Data are represented as mean ± SEM. *P < 0.05, **P < 0.01 compared with the corresponding sham-operated group. ∆P < 0.05, ∆∆P < 0.01 compared with the Hab-Sta sham-operated group.

**Figure 3:**
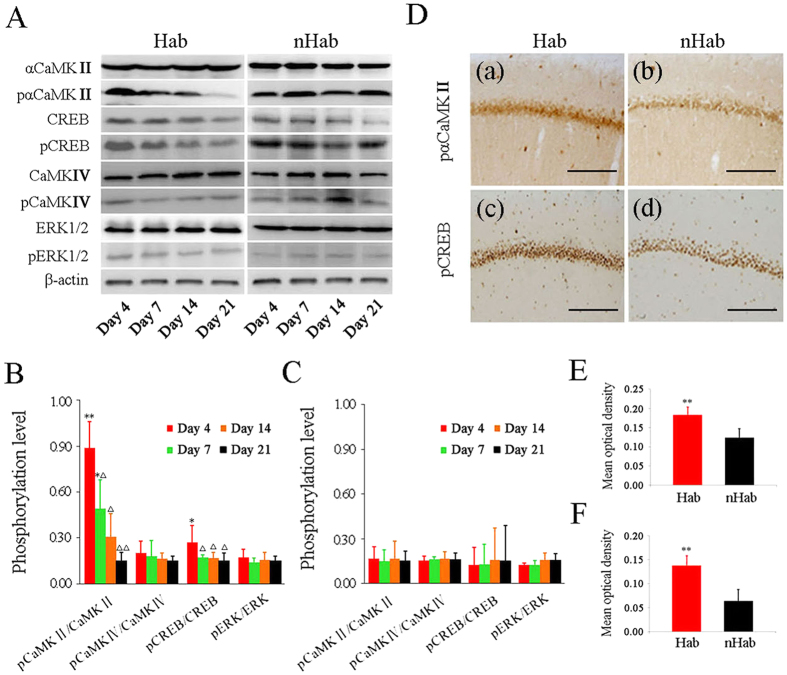
Changes in kinases activity in the hippocampal CA1 subregion in Hab and nHab animals during “internal model” retention period. Representative images of western blot analysis (**A**) and statistical plot for the ratio of pαCaMKII/αCaMKII, pCREB/CREB, pCaMKIV/CaMKIV and pERK1/2/ERK1/2 in animals exposed to Rot on Day 4, 7, 14 and 21 after Hab (**B**) and nHab (**C**) treatment period. *P < 0.05, **P < 0.01 compared with the corresponding nHab group. ∆P < 0.01, ∆∆P < 0.001 compared with the Hab group on Day 4. Representative pictures of immunohistochemistry staining (**D**) for pαCaMKII (a, b) and pCREB (c, d) in animals exposed to Rot on Day 4 after Hab and nHab treatment period. The statistical plots for the mean optical density are shown in (**E**) and (**F**). Bar = 200 μm; *P < 0.05, **P < 0.01 compared with the nHab group.

Histological analysis showed that ibotenic acid almost completely removed the cells in the CA1 (2.5~4.5 mm caudal to bregma; mean lesion volume = 84.2%; Fig. 2A1), in the CA2/3 (2.3~4.3 mm caudal to bregma; mean lesion volume = 87.5%; Fig. 2B1), while colchicine induced almost complete cell lesions in the DG (2.5~5.3 mm caudal to bregma; mean lesion volume = 81.8%; Fig. 2C1) with minimal damage to the surrounding tissues. In the Hab-Rot animals, bilateral hippocampal CA1 lesion significantly increased defecation responses (F_(5, 29)_ = 11.518, P < 0.001) and the time to traverse the horizontal balance beam (F_(5, 29)_ = 5.363, P < 0.01) after Rot on Day 4 during retention period compared with the sham controls (post hoc: P < 0.01; Fig. 2A2 and A3). The CA1-lesioned animals also showed a decrease in total distance travelled (F_(5, 29)_ = 14.012, P < 0.001) and activity frequency (F_(5, 29)_ = 15.650, P < 0.001) in an open field (P < 0.01; Fig. 2A4 and A5). In contrast, the sham-operated Hab-Rot animals showed no significant behavioral alterations compared with sham-operated Hab-Sta controls (P > 0.05). There was also no significant difference between CA1-lesioned and sham-operated animals receiving Sta treatment during retention period (P > 0.05). Bilateral hippocampal CA1 lesion also had no effect on behavioral responses in nHab animals which showed significant MS responses (P < 0.001) compared with sham-operated Hab-Sta controls (Fig. 2A2–A5). Likewise, bilateral DG lesion also elicited significant defecation responses (F_(5, 29)_ = 28.104, P < 0.001) and balance disorders (F_(5, 29)_ = 11.453, P < 0.001) in Hab-Rot animals (LSD post hoc: P < 0.05 or 0.01; Fig. 3B2 and B3) but not in Hab-Sta animals (P > 0.05) compared with corresponding sham-operated controls. However, regardless of whether the animals received Hab training or not, spontaneous locomotor activity (total distance travelled: F_(5, 29)_ = 127.173, P < 0.001; activity frequency: F_(5, 29)_ = 31.652, P < 0.001) were significantly inhibited in DG-lesioned animals compared with sham-operated controls (LSD post hoc: P < 0.05 or 0.01; Fig. 3B4 and B5). In addition, bilateral CA2/3 lesion had no significant effect on defecation response, balance coordination and total distance travelled (P > 0.05), but decreased activity frequency in Hab animals (P < 0.05) on Day 4 during retention period compared with corresponding sham-operated animals. The Rot stimulation induced MS responses including defecation response (F_(5, 29)_ = 11.807, P < 0.001), balance disorder (F_(5, 29)_ = 22.083, P < 0.001) and hypoactivity (total distance travelled: F_(5, 29)_ = 4.522, P < 0.05; activity frequency: F_(5, 29)_ = 4.522, P < 0.01) in nHab groups (LSD post hoc: P < 0.05 or 0.01) but not in CA2/3-lesioned Hab-Rot animals (P > 0.05) compared with sham-operated Hab-Sta controls.

### CaMKII and CREB activity increased in rat CA1 subregion during “internal model” retention period

Figure 3 shows the changes in kinases activity in hippocampal CA1 subregion throughout the “internal model” retention period. Western blot analysis showed that Hab training significantly increased the phosphorylation of αCaMKII (Hab effect: F_(1, 32)_ = 16.372, P < 0.001; Day effect: F_(3, 32)_ = 10.054, P < 0.001; Hab × Day interaction: F_(3, 32)_ = 8.827, P < 0.001) and CREB (Hab effect: F_(1, 32)_ = 14.019, P < 0.01; Day effect: F_(3, 32)_ = 5.765, P < 0.01; Hab × Day interaction: F_(3, 32)_ = 5.575, P < 0.01) after Rot on Day 4 compared with the phosphorylation levels on Day 7, 14 and 21 (LSD post hoc: P < 0.01 or 0.001, Fig. 3A and B). The phosphorylation levels of αCaMKIIon Day 4 and 7 and CREB on Day 4 were also significantly higher in the Hab animals than the corresponding nHab controls (P < 0.05 or 0.01). Hab training had no significant effect on the phosphorylation of CaMKIV (Hab effect: F_(1, 32)_ = 0.422, P > 0.05; Day effect: F_(3, 32)_ = 0.289, P > 0.05; Hab × Day interaction: F_(3, 32)_ = 1.033, P > 0.05) or ERK-1/2 (Hab effect: F_(1, 32)_ = 2.728, P > 0.05; Day effect: F_(3, 32)_ = 1.083, P > 0.05; Hab × Day interaction: F_(3, 32)_ = 1.023, P > 0.05) during retention period (Fig. 3A and B). There was also no difference in the phosphorylation of these four kinases among nHab groups (post hoc: P > 0.05; Fig. 3A and C). Similar to the results of western blot analysis, immunohistochemistry staining confirmed that Hab training significantly increased the expression of pαCaMKII (P < 0.01; Fig. 3D(a) and E) and pCREB (P < 0.01; Fig. 3D(c) and F) in the CA1 subregion on Day 4 of the retention period compared with nHab group (Fig. 3D(b,d)).

### Inhibition of CaMKII but not ERK1/2 in rat CA1 subregion impaired “internal model” retention

**Figure 4:**
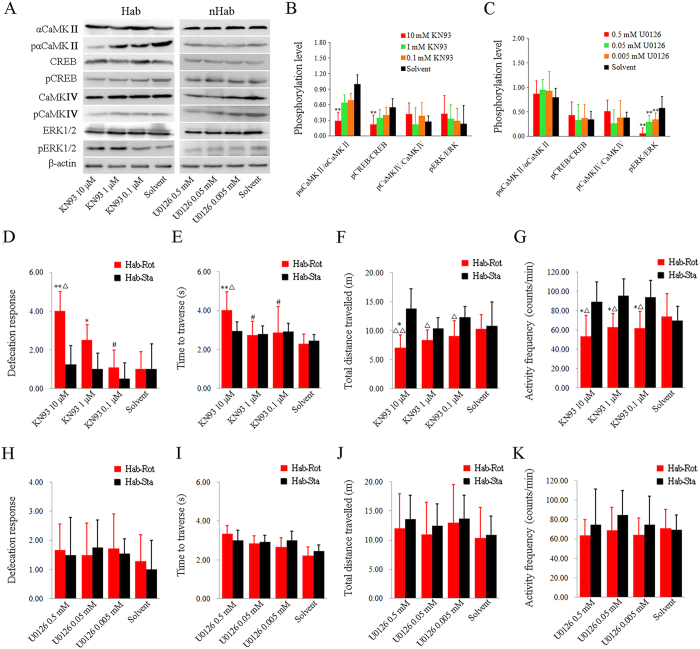
Effects of CaMKII or ERK1/2 inhibition in hippocampal CA1 subregion on MS-related behavioral responses during “internal model” retention period. Representative images and statistical plot of data of western blot analysis for the ratio of pαCaMKII/αCaMKII, pCREB/CREB, pCaMKIV/CaMKIV and pERK1/2/ERK1/2 after KN93 (**A** and **B**) or U0126 (**A** and **C**) injection into hippocampal CA1 subregion. Defecation responses (**D** and **H**), time to traverse the balance beam (**E** and **I**), as well as total distance travelled (**F** and **J**) and activity frequency (**G** and **K**) in an open field were analyzed after Rot or Sta treatment on Day 4 during retention period in animals receiving Hab training. Data are represented as mean ± SEM. *P < 0.05, **P < 0.01 compared with the corresponding solvent control group. ∆P < 0.05, ∆∆ P < 0.01 compared with the corresponding Hab-Sta group. ^#^P < 0.05 compared with the Hab-Rot animals receiving 10 μM KN93 injection.

Intra-CA1 application of the CaMKII inhibitor KN93 significantly inhibited the phosphorylation of αCaMKII (F_(3, 28)_ = 4.085, P < 0.05) and CREB (F_(3,28)_ = 5.200, P < 0.01) in Hab-Rot animals on Day 4 of the “internal model” retention period (Fig. 4A and B). Administration of KN93 at a dose of 10 μM significantly decreased both pαCaMKII/αCaMKII and pCREB/CREB compared with the solvent controls (P < 0.01) and the 0.1 μM KN93 group (P < 0.05). No significant effect of KN93 on the phosphorylation of CaMKIV (F_(3, 28)_ = 1.103, P = 0.364) and ERK1/2 (F_(3, 28)_ = 0.363, P = 0.780) was observed (Fig. 4A and B). Furthermore, intra-CA1application of KN93 at 10 μM and 1 μM significantly increased the defecation responses on Day 4 of the “internal model” retention period in the Hab-Rot animals compared with the solvent controls (one-way ANOVA: F_(7, 63)_ = 4.185, P < 0.01; post hoc: P < 0.01 and 0.05) and the corresponding Hab-Sta animals (P < 0.05; Fig. 4D). Animals injected with 10 μM KN93 also showed an increase in defecation responses compared with those receiving 0.1 μM KN93 (P < 0.01). KN93 at 10 μM also prolonged the time to traverse the balance beam in the Hab-Rot animals compared with the solvent controls (P < 0.01), the 0.1 μM KN93 and the 1 μM KN93 groups (P < 0.05) as well as the corresponding Hab-Sta animals (Fig. 4E). In the open field test, the KN93 at all three doses significantly decreased total distance travelled and activity frequency in the Hab-Rot animals compared with corresponding Hab-Sta controls (P < 0.05 or 0.01; Fig. 4F and G). The Hab-Rot animals injected with 10 μM KN93 also had lower spontaneous locomotor activity levels compared with the solvent controls (P < 0.05). In the Hab-Sta animals, no significant effect of KN93 on these behavioral responses was observed compared with the vehicle controls.

Intra-CA1 application of ERK1/2 inhibitor U0126 at all three doses significantly decreased ERK1/2 activity compared with the solvent controls (F_(3,28)_ = 9.278, P < 0.001; LSD post hoc: P < 0.01) in the Hab-Rot animals (Fig. 4A and C). In contrast to KN93, U0126 had no significant effect on defecation responses (F_(3,28)_ = 0.143, P = 0.933; Fig. 4H), balance beam performance (F_(3,28)_ = 0.599, P > 0.05; Fig. 4I) and spontaneous locomotor activity (distance travelled: F_(3,28)_ = 0.870, P > 0.05; activity frequency: F_(3,28)_ = 0.669, P > 0.05; Fig. 4J and K).

### CaMKII and CREB activity did not change in the CA1 of the *het/het* mice on the day of “internal model” retention period

**Figure 5:**
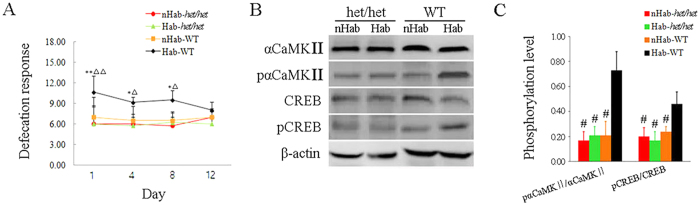
Changes in CaMKII and CREB activity in hippocampal CA1 subregion in Hab and nHab *het/het* mice on Day 4 of “internal model” retention period. (**A**) Defecation responses in the *het/het* mice and the wild-type (WT) mice receiving Hab or nHab treatment during “internal model” acquisition period. Representative images of western blot analysis (**B**) and statistical plot (**C**) for the ratio of pαCaMKII/αCaMKII and pCREB/CREB in the *het/het* mice and the WT mice re-exposed to Rot on Day 4 after Hab or nHab treatment period. *P < 0.05, **P < 0.01 compared with the corresponding nHab-WT animals. ∆P < 0.05, ∆∆ P < 0.01 compared with the corresponding Hab-*het/het* animals. ^#^P < 0.01 compared with the Hab-WT animals.

Two-way ANOVA analysis showed significant effects of Hab treatment (F_(1,80)_ = 15.000, P < 0.001), vestibular deficit (F_(1,80)_ = 52.948, P < 0.001) and Hab treatment × vestibular deficit interaction (F_(1,80)_ = 15.143, P < 0.001) on defecation responses during “internal model” acquisition period. Defecation responses were significantly increased in the WT mice after 1, 4 and 8 Rot sessions compared with the corresponding nHab controls and Hab-*het/het* animals (LSD post hoc: P < 0.05 or 0.01; Fig. 5A). No significant difference was observed between the Hab-WT and the nHab-WT groups after 12 sessions of rotation, indicating the acquisition of a novel “internal model”. In contrast, the Hab-*het/het* mice showed no significant defecation response compared with the nHab-WT and the nHab-*het/het* group during the whole training period (Fig. 5A). Western blot analysis showed that the Hab-WT mice had higher levels of pαCaMKII/αCaMKII (Hab treatment: F_(1, 20)_ = 46.993, P < 0.001; vestibular deficit: F_(1, 20)_ = 46.414 P < 0.001; Hab treatment × vestibular deficit interaction: F_(1, 20)_ = 45.468, P < 0.001) and pCREB/CREB (Hab treatment: F_(1, 20)_ = 9.216, P < 0.01; vestibular deficit: F_(1, 20)_ = 45.706 P < 0.001; Hab treatment × vestibular deficit interaction: F_(1, 20)_ = 10.329, P < 0.01) than the Hab-*het/het*, the nHab-*het/het* and the nHab-WT animals (LSD post hoc: P < 0.001; Fig. 5B and C) after Rot on Day 4 of the “internal model” retention period. No significant difference was observed in CaMKII and CREB activity among Hab-*het/het*, nHab-*het/het* and nHab-WT animals (LSD post hoc: P > 0.05).

## Discussion

Our previous studies have demonstrated that MS-related behavioral responses including gastrointestinal symptom (defecation) and vestibular-related disorders (balance disturbance and hypoactivity) are valid assays for MS susceptibility and MS habituation in rodents^11^,^12^,^27^. The current study confirmed that repeated passive motion stimulation induced a progressive and almost linear decrease in these behavioral responses which gradually reappeared at least 4 days after the habituation training stopped. This form of vestibular-associated behavioral plasticity seems to involve the nonassociative learning process which is usually characterized by the alleviation of responses after repeated exposure to a given stimulus^28^. In addition to motion challenges, repeated galvanic vestibular stimulation also can induce short-term (fast) habituation in galvanic-induced body sway within minutes in humans^29^. Nevertheless, MS susceptible subjects who can not habituate effectively to the rotatory and vehicle motion stimuli may still habituate normally to galvanic vestibular stimulation^30^. These results suggest that the MS habituation might be irrelevant to the sensory reweighting process of vestibular adaptation. In addition, there are several lines of evidence supporting that the hypothalamic-pituitary-adrenal (HPA) axis might contribute to the nonassociative learning about stress after chronic intermittent exposure to a variety of stressors^31^. However, the temporal changes of blood stress hormones did not synchronize those of motion-induced behavioral responses^32^. For example, 2 sessions of body rotation in humans sufficiently induced habituation in the HPA system, but the nausea responses habituated almost after 4 sessions of rotation^33^. The present study also found that rotation stimulation did not induce MS-related defecation responses in genetic otolith deficit mice during habituation training period. These results suggested that vestibular activation but not general stress contributed to the MS-related behavioral responses in normal wild-type animals.

Electrophysiological studies have demonstrated that dorsal hippocampus is essential for spatial cognition and memory and nonspatial object memory in rodents^34^,^35^. A functional magnetic resonance imaging (fMRI) study showed that hippocampal activation significantly correlated with implicit motor sequence learning in humans^36^. In rodents, hippocampectomy impairs the memory of recently, but not remotely, acquired trace eyeblink conditioned responses^37^. It suggests that hippocampus contribute to the formation and/or storage of nondeclarative memory in both humans and animals. The present study found that chemical lesion of the hippocampal CA1 region but not the CA2/3 region reproduced MS-related behavioral responses in habituated animals during “internal model” retention period. However, the limitations of the lesion technique should also be considered. For example, the lesions may encompass the passing fibers and can produce a general deficit in behavioral performance. Based on the fact that CA1 lesion did not affect the behaviors in either the non-habituated animals exposed to rotation or the habituated animals exposed to static treatment during retention period, we presume that CA1 lesion in the habituated animals might interfered “internal model” retention which still remained intact in sham-lesioned controls. In contrast, chemical lesion of DG area also reproduced defecation and balance disturbance in habituated animals, and generally inhibited spontaneous locomotion in both habituated and non-habituated ones, suggesting that DG-lesion might induce general behavioral inhibition and/or facilitatory effects in rats^38^.

Based on the behavioral results, we consider that the neural storage of the novel passive motion pattern might refer to the CA1-dependent nondeclarative memory system which provides varieties of unconscious ways of responding to the world^39^. Our study further revealed that CaMKII and CREB activity were initially enhanced and then gradually decreased in habituated animals during “internal model” retention period. It is well known that the αCaMKII homomers can be formed in excitatory hippocampal pyramidal neurons through dendritic αCaMKII localization after neuronal stimulation^40^. Previous studies confirmed that αCaMKII autophosphorylation at the threonine residue 286 (T286) is crucial for the induction of NMDAR-dependent LTP in the hippocampal CA1^20^,^41^. Our study showed that the phosphorylation of αCaMKII was increased after rotation in the CA1 region on Day 4 during retention period. This result was consistent with a previous observation showing that αCaMKII phosphorylation was persistently enhanced for at least 8 h after the induction of LTP^42^. It is widely accepted that the CaMKII signaling pathway is critical for both declarative and non-declarative memory formation. For example, T286A mutation in αCaMKII severely impaired in the acquisition of spatial cognition, while overexpression of hippocampal CaMKII can improve performance in the Morris water maze task^41^,^43^. The T286A mutation in αCaMKII also impaired hippocampus-dependent contextual fear conditioning but not amygdala-dependent cued fear conditioning after using multiple tone–shock pairings training^44^. Meanwhile, the race eyeblink conditioning after multiple training trials did not require autophosphorylation of αCaMKII in mice^45^, while the formation of memory to a novel environment depends on activation of NMDAR and CaMKII^46^. It seems that CaMKII mainly contribute to the formation and/or storage of learning and memory established by spatial information which can be detected by visual and vestibular organs. Our study showed that CaMKII phosphorylation did not change in non-habituation animals, suggesting that the increased αCaMKII autophosphorylation in habituated animals might be due to the re-exposure to passive motion stimulation but not to the familiar context during retention period. In addition, as we know that CaMKIV and ERK1/2 is also important for learning and memory. CaMKIV knockout impaired memory of both eyeblink conditioning and contextual fear conditioning in mice^47^,^48^. Contextual fear conditioning induces ERK1/2 activation in the hippocampus after training, while post-training administration of the blocker of ERK1/2 cascade impairs fear memory formation^49^. It is noteworthy that there is cross-talk between the CaM kinase cascade and ERK signaling through activation of downstream CREB during memory consolidation^50^. However, our study showed that habituation training did not change CaMKIV or ERK1/2 activity but significantly increased CREB activity during “internal model” retention period. Furthermore, intra-CA1 injection of KN93, a selective CaMKII blocker, significantly induced MS-related behavioral responses during “internal model” retention period, suggesting that the recall of passive motion memory was hindered by CaMKII inhibition. However, KN93 is also thought to have potent effects on P2 × 7 receptors, K^+^ and Ca^2+^ channels and other members of the CaMK as well^51^. The present study confirmed that KN93 administration inhibited the phosphorylation of both CaMKII and CREB but had no effect on CaMKIV and ERK1/2 activity, indicating that KN93 suppresses both the phosphorylation of CaMKII and the phosphorylating activity of CaMKII in our study^52^. In contrast, inhibition of the ERK1/2 signaling by U0126 did not interfere with “internal model” retention. These results suggested that the CaMKII-CREB pathway, but not ERK1/2 cascade, might contribute to the storage of “internal model” derived from the repeated passive motion stimulation.

Electricophysiology studies showed that bilateral lesions of the peripheral vestibular system completely abolished location-related firing and theta rhythm generation in CA1 neurons^53^,^54^. Bilateral vestibular deafferentation significantly decreased basal dendritic length in the rat hippocampal CA1 region^55^. Damage to the peripheral vestibular system also induced changes in expression of hippocampal NMDA receptor subunits, and reduced spatial training-associated increases in glutamate receptor and αCaMKII expression in rats^56^,^57^,^58^. A fMRI combined with extracellular electrophysiology study found that electrical stimulation of the vestibular organ significantly activate the CA1 area in rats^59^. Our study revealed that CaMKII or CREB activity did not increase in the CA1 after habituation training in the genetic otolith deficit *het/het* animals. This observation echoes the results of the pharmacological experiments and provides the evidence that passive motion information detected by the vestibular system might be transferred to the hippocampal CA1 region. The activation of CaMKII or CREB after rotation during “internal model” retention period might be the outcomes of the re-call of the vestibular memory established during “internal model” acquisition period.

In summary, repeated passive motion stimulation induced MS-habituation in rats, indicating the establishment of a novel “internal model”. The habituated animals showed resistance to MS when they were re-exposed to rotation at least within 4 days after the habituation training stopped. Chemical lesion of the hippocampal CA1 region reproduced MS-related behavioral responses on the day of “internal model” retention. Habituation training lead to the phosphorylation of CaMKII and CREB but had no effect on the phosphorylation of CaMKIV and ERK1/2 in hippocampal CA1 region after rotation on the retention day. Inhibition of CaMKII but not ERK1/2 activity elicited MS-related behavioral responses in habituated animals indicating the interference with neuronal storage of the “internal model”. In genetic otolith deficit *het/het* mice, rotation stimulation failed to induce MS-related behavioral responses during “internal model” acquisition period, and the CaMKII and CREB were not activated in the CA1 region after rotation during the retention period. These results suggested that the sensory of passive motion detected by the vestibular system might be processed and stored in hippocampus which can produce novel anticipated sensory patterns known as the “internal model”. The CaMKII-CREB signaling pathway might contribute to the formation and/or storage of the “internal model” in the hippocampal CA1 region after repeated passive motion exposure.

## Methods

### Animals and ethnics

Male adult Sprague–Dawley rats (300–350 g) were purchased from Shanghai Laboratory Animal Center. The male adult (29–32 g) *het/het* mice (C57BL/6J-Nox3*het)* were obtained from the Jackson Laboratory. Animals were individually housed under a 12 h light: 12 h dark cycle with free access to food and water. All animal protocols and procedures complied with the Guide for the Care and Use of Laboratory Animals (US National Research Council, 1996) and were approved by the Ethics Committee for Animal Experimentation of the Second Military Medical University (Shanghai, PR China). All surgeries were performed under sodium pentobarbital anesthesia (40 mg/kg or 80 mg/kg for rats, 70 mg/kg or 120 mg/kg for mice, i.p.).

### Rotation device and procedures

The rotation device and detailed rotation methods were described previously^11^. Briefly, the animals were placed in plexiglass containers with the long axis of the body perpendicular to the horizontal rotation rod to stimulate the otolith organs (anterier-posterier and vertical direction) during rotation. The device started to rotate in a clockwise direction at 16°/s^2^ to reach an angular velocity of 120°/s and then began to decelerate at 48°/s^2^ to reach 0°/s. After a 1 s pause, the container continued to rotate in a counterclockwise direction in the same manner as above. The clockwise-pause-counterclockwise cycle lasted approximately 21 s. Each session of rotation lasted for 2 h. Before the experiment, animals were pre-adapted to the equipment for 2 h per day for 3 days. These procedures were performed during 8:00–12:00 p.m. in complete darkness at 22 °C.

### Experiment design and procedures

#### Experiment 1

Establishment of “internal model” acquisition model: 96 animals were used and randomly divided into four habituation (Hab) groups receiving 1, 4, 7 or 10 rotation (Rot) sessions on a daily basis (2 h/d), respectively, and four corresponding non-habituation (nHab) groups receiving static (Sta) treatment without rotation^11^ (n = 12 in each group). Establishment of “internal model” retention model: another 80 rats were used and randomly divided into four Hab-Rot and four Hab-Sta groups (n = 10 in each group). They were re-exposed to 2 h Rot or Sta on Day 4, 7, 14 or 21 after a 10-day Hab training stopped. Immediately after the last Rot or Sta session, half animals in each group were randomly selected for balance beam test and the remaining half received open field test. The defecation response was recorded in all animals.

#### Experiment 2

Ninety rats were randomly divided into three groups: Hab-Rot group, Hab-Sta group and nHab-Rot group. Animals in each group were assigned to three lesion subgroups receiving a lesion to CA1, CA2/3, or the dentate gyrus (DG), or three corresponding sham operation subgroups immediately after the last session of Hab or nHab treatment (n = 5 in each subgroup). Four days later, the animals were re-exposed to Rot or Sta treatment and MS-related behavioral responses were evaluated.

#### Experiment 3

Eighty animals were divided into four Hab groups or four nHab groups (n = 10 in each group) and were subjected to a 2 h Rot on Day 4, 7, 14 or 21, respectively during retention period. The phosphorylation of αCaMKII, CaMKIV, CREB and ERK1/2 in CA1 region was tested using western blot analysis in half of the animals in each group after the Rot treatment (n = 5). The results were further verified in another half using immunohistochemistry (n = 5).

#### Experiment 4

One hundred and sixty animals were used and divided into four Hab groups or four nHab groups (n = 10 in each group) receiving intra-CA1 injection of CaMKII inhibitor KN93 (0.1, 1 or 10 μM; Merk Millipore, USA) or solvent, and additional four Hab groups or four nHab groups (n = 10 in each group) receiving intra-CA1 injection of ERK1/2 inhibitor U0126 (0.005, 0.05 or 0.5 mM; Selleck, Houston, USA) or solvent on Day 4 during retention period. Animals were re-exposed to Rot 30 min after injection. Then the behavioral responses were observed and the CaMKII, CaMKIV, CREB and ERK1/2 phosphorylation level in CA1 region was tested.

#### Experiment 5

In this part, 12 *het/het* mice and 12 wild-type (WT) littermates were used and subdivided into four groups: Hab-*het/het*, Hab-WT, nHab-*het/het* and nHab-WT (n = 6 in each group). The “internal model” acquisition was judged when no difference in defecation responses between Hab-WT and nHab-WT animals was observed. The phosphorylation of CaMKII and CREB in the CA1 region was examined after 2 h Rot on Day 4 during retention period.

## MS symptom assessment

### Defecation and balance beam test

Immediately after each Rot or Sta treatment, animals were taken out of the plexiglass containers of the rotation device. The number of fecal granules deposited by each animal was then counted. The balance performance was assessed by measuring the time that each rat spent to traverse an elevated (90 cm) narrow wooden beam (2.5 × 150 cm) and enter a black plastic box (15 cm × 15 cm × 8 cm) at the opposite end. Before testing, each animal was trained daily for 5~7 consecutive days in order to achieve a stable performance on the beam. Three testing trials were performed with a 60 s rest and the average was used for statistical analysis.

### Spontaneous locomotor activity test

Spontaneous locomotion was measured by an animal behavior test system (RD1112-IFO-R-4, Mobiledatum, Shanghai, China) consisting of a dark rectangular chamber (50 × 50 × 60 cm) with the floor marked with a 16 × 16 grid. The testing was conducted in a soundproof room. The animal was placed in the center of the chamber and left undisturbed for 5 min when the behavior and locomotion tracking were recorded. The total distance travelled (m) and activity frequency (counts/min) were measured with a commercially available software (EthoVision XT 8.5, Noldus, Netherlands)^26^.

### Hippocampal lesion and drug injection

Bilateral hippocampal lesions were produced by microinjections of 1% ibotenic acid (Sigma Aldrich, St. Louis, USA) within dorsal CA1 (3 injection sites: AP −3.6 mm, ML 1.0 mm, 2.0 mm, 3.0 mm relative to bregma; DV 1.9 mm ventral to the dura; 0.10~0.15 μl) and dorsal CA2/3 (3 injection sites: AP −2.8 mm, −3.3 mm and −4.1 mm, ML 3.0 mm, 3.4 mm and 4.2 mm relative to bregma; DV 3.2 mm, 3.2 mm and 3.3 mm ventral to the dura; 0.05~0.15 μl) and by injection of 2.5 μg/μl colchicine (Aladdin, Hangzhou, China) within dorsal DG (2 injection sites: AP −2.8 mm and −4.3 mm, ML 1.4 mm and 2.3 mm relative to bregma; DV 3.4 mm and 3.0 mm ventral to the dura; 0.06 μl) through a glass micropipette (tip caliber 50–100 μm) attached to a Hamilton microsyringe (1 μl). The sham-operated animals received the injection of solvent following the same procedures.

For drug injection, two 23-gauge stainless steel guide cannulaes (RWD Life Science, Shenzhen, China) were implanted into bilateral CA1 regions (3.6 mm caudal to bregma, 2.0 mm lateral to midline, and 1.8 mm ventral to the dura). During drug administration, the guide cannula was fitted with a 30-gauge stainless steel injection needle that extended 0.5 mm beyond the tip of the guide cannula. Drug solution (0.5 μl) was injected by pressure within 10~15 s and the needle was retained for 5 min in each injection site. All animals received antibiotics penicillin (400000 U/kg, i.p.) and analgesics ibuprofen (30 mg/kg, in the drinking water) once a day for 3 days after lesion and implantation surgery.

### Tissue preparation

In western blot experiments, animals were perfused transcardially with chilled saline. The brains were immediately dissected out and cooled in iced saline for 1 min. A blunted 22 or 26 gauge needle was used to dissect bilateral CA1 subregion from thick sections in rat or mouse. The bilateral dissected tissues were pooled as one sample and the remaining parts of sections were used for Nissl staining to verify the location of dissection.

In the histological experiments, rats were perfused transcardially with 100 ml chilled saline, followed by perfusion with 500 ml 4% paraformaldehyde. The brain blocks were postfixed with 4% paraformaldehyde at 4 °C for 1 h and then placed in 30% sucrose overnight at 4 °C before made into 20 μm-thick sections. Seventeen sections were collected for evaluation of hippocampal lesions in each animal after Nissle staining. The volume of the hippocampal lesion was expressed as percentage of the mean volume of hippocampal subregions in the sham-operated group^60^. In the immunohistochemical experiments, one out of every 3 consecutive sections was selected for immunostaining.

### Western blot analysis

The tissue sample was abraded and lysed and was then centrifuged at 10,000 g for 5 min at 4 °C. Protein (50 μg per lane) from each sample was loaded on a 10% sodium dodecyl sulfate-polyacrylamide gel, electrophoresed, and transferred onto nitrocellulose membrane (Millipore Corp, Bedford, MA, USA). The membranes were then incubated overnight at 4 °C with the primary antibodies against pαCaMKII, αCaMKII, pCREB, CREB, pαCaMKIV, αCaMKIV, pERK1/2 or ERK1/2 (1:1000; Millipore Crop, Bedford, MA, USA) in TBST with 1% BSA. After washing with TBST, the membranes were incubated for 2 h with peroxidase-labeled secondary antibodies (1:5000, Jackson, West Groove, USA) at room temperature. The bands were then visualized and analyzed by the Gel Doc image system (BioRad, San Diego, USA). Signal intensities were normalized against the internal control (β-actin). The phosphorylation levels for the kinases were calculated by normalizing the levels of phosphorylated protein to the total protein levels.

### Immunohistochemistry

Tissue sections were washed in 0.01 M phosphate-buffered solution (PBS) and incubated in a rabbit anti-pαCaMKII IgG or goat anti-pCREB IgG (1:100) for 24 h at 4 °C. After washing in PBS, the sections were incubated in biotinylated goat anti-rabbit IgG (1:200; Vector Laboratories, Burlingame, USA) for 4 h. The immunolabeling was visualized using ABC method according to the manufacture’s instruction (Vector Laboratories, Burlingame, USA). Optical density of pαCaMKII or pCREB was measured using the Image ProPlus 6.0 software (Media Cybernetics, Silver Spring, USA).

### Statistical analysis

Statistical analysis was performed using the SPSS v13.0 statistical program. One-way ANOVA combined with LSD post hoc analysis was performed in Experiment 1, 3 and 4. Two-factorial ANOVA was also used in Experiment 2 and 5. Fisher’s LSD post hoc test was performed when a significant main or interaction effect was obtained. All data were expressed as mean ± SEM. Statistical significance was set at P < 0.05.

## Additional Information

**How to cite this article:** Wang, J. *et al*. Storage of passive motion pattern in hippocampal CA1 region depends on CaMKII/CREB signaling pathway in a motion sickness rodent model. *Sci. Rep.* **7**, 43385; doi: 10.1038/srep43385 (2017).

**Publisher's note:** Springer Nature remains neutral with regard to jurisdictional claims in published maps and institutional affiliations.
